# Advocacy to improve food security in remote Aboriginal and Torres Strait islander communities in Australia: measuring the impact of message frames

**DOI:** 10.1017/S1368980026102845

**Published:** 2026-06-03

**Authors:** Madura Katta, Michael Waller, Megan Ferguson, Kani Thompson, Caroline Deen, Emma Tonkin, Julie K. Brimblecombe, Ellie Chan, Amanda Lee, Emma Chappel, Katherine Cullerton

**Affiliations:** 1 School of Public Health, The University of Queensland, Australia; 2 Menzies School of Health Research, Charles Darwin University, Australia; 3 Apunipima Cape York Health Council, Australia; 4 Sydney School of Health Sciences, The University of Sydney, Australia; 5 Department of Nutrition, Dietetics and Food, Monash University, Australia; 6 School of Public Health, Menzies School of Health Research, Australia; 7 Central Australian Aboriginal Congress, Australia

**Keywords:** food security, food and nutrition policy, framing, Aboriginal and Torres Strait Islander peoples, remote communities

## Abstract

**Objective:**

To examine whether different message frames increase public support for a community-identified policy solution to improve food security in remote Aboriginal and Torres Strait Islander communities in Australia.

**Design:**

An online experimental study in which participants were randomly assigned to one of three message frames: human rights, fairness or community-identified solution or a control condition. Logistic regression models were used to assess associations between message frames and support for an electricity subsidy policy. Interaction analyses explored whether participant characteristics moderated these effects.

**Setting:**

Australia.

**Participants:**

A nationally representative sample of 1555 Australian adults recruited through an online panel.

**Results:**

Message framing had limited overall impact on policy support. The human rights frame generated the highest support for the proposed subsidy, while the fairness frame generated the lowest; however, these differences were modest. We found that regardless of frame used, Greens supporters, those with postgraduate degrees or those who watched the news more frequently, tended to have higher levels of agreement with the policy solution.

**Conclusion:**

Although message framing is widely used in advocacy to increase policy support, its impact in this context appears constrained. These findings underscore the complexity of shifting public attitudes toward policies benefiting structurally disadvantaged communities and suggest that more targeted, audience-specific framing may be necessary. Further research should explore why certain frames underperform among particular groups and examine how agreement and emotional engagement jointly shape support for policy change.

## Introduction

Advocacy is an essential element of public health, focussed on promoting illness prevention and public policy to improve the health and well-being of individuals and communities. To gain the public and political support that is necessary for policy change, public health advocates use a variety of strategies including media engagement, community mobilisation and challenging corporate narratives^(1)^.

Effectively communicating the efficacy and expected outcomes of proposed policy actions is key to the success of most advocacy strategies. Public health advocates are increasingly using framing when developing messages, to draw attention to problems and gain public and policymaker support for proposed solutions^(2)^. Framing is ‘the process by which people develop a particular conceptualisation of an issue or reorient their thinking about an issue’, which shapes their understanding of problems, evidence and solutions^(3)^. Effective framing often resonates with people’s core values and worldviews, making it a powerful tool in public health advocacy^(2)^.

Ensuring food security for all is an increasing public health challenge in Australia and globally^(4,5)^. Food security exists when people have ‘physical and economic access to sufficient safe and nutritious food that meets dietary needs and food preferences for an active and healthy life’^(6)^. Food insecurity is associated with an increased risk of chronic conditions including diabetes, CVD, anxiety and depression in adults^(7,8)^. For children, the impacts are profound, affecting physical, cognitive and behavioural development^(9)^.

In Australia, as in many countries, certain population groups disproportionally experience food insecurity due to social, geographic and economic inequities^(10)^. While the overall prevalence of food insecurity in Australia is estimated at 4 to 5 %^(11)^, the prevalence is significantly higher for Aboriginal and Torres Strait Islander peoples. In 2023, two out of five Aboriginal and Torres Strait Islander households (41 %) experienced food insecurity due to insufficient funds for food at some time in the previous 12 months, with the proportion even higher in remote areas (51 %)^(12)^.

Aboriginal and Torres Strait Islander peoples maintained food security through their detailed knowledge of Country, and diverse dietary patterns of fresh plant and animal foods, over millennia^(13)^. However, colonisation and systemic racism, including the forcible takeover of the land, displacement of people, disruption of knowledge systems and introduction of western foods, have impacted on food security, especially in remote areas^(14,15)^.

Communities have identified many and varied solutions to address food insecurity, including both community-led initiatives and recommendations for policy change^(14,16)^. While there has been some community-level action, government responses have been limited^(14,16)^. In remote communities, the challenges to food security are manifold: restricted access to land and water ways, lack of resources for traditional food procurement, inadequate income and limited employment opportunities to increase income, high food prices driven by long and fragile supply chains, and housing that is unsuitable for remote conditions^(14)^. Impacted by both income and housing and infrastructure challenges, energy insecurity is an issue which is linked to food security. In many remote communities, the cost of pre-paid energy competes with household income available for food, and unreliable power supply compromises the usability of household resources such as refrigerators and stoves for food storage and preparation^(17–19)^. To date, calls for federal government action to address energy security in remote Aboriginal and Torres Strait Islander communities have been largely unaddressed.

This study was designed to explore the potential of message frames to increase the saliency of policy action to address food and energy insecurity in remote communities in Australia. Specifically, we investigated whether different message frames increase individuals’ support for an electricity subsidy and whether political party (voting) preference or other sociodemographic factors affect this support.

While previous studies have examined the framing of *existing* policies^(20–22)^, to our knowledge there has been no examination of message frames to increase public support for *new* policies aimed at improving health and well-being among Aboriginal and Torres Strait Islander peoples in remote communities.

## Methods

### Study design

We conducted a cross-sectional survey that included quantitative and free-text responses. Approval for the research was granted by the Research Governance Committee of Apunipima Cape York Health Council and Central Australian Aboriginal Congress Board. Ethics approval was granted by the Central Australian Human Research Ethics Committee (CA-20-3701) and The University of Queensland Human Research Ethics Committee B (2020000636).

### Development of message frames

This study was part of a comprehensive Remote Food Security Project that was developed in response to a call to action and position statements from Aboriginal Community Controlled Health Organisations; Apunipima Cape York Health and Central Australian Aboriginal Congress. It aimed to determine community-led solutions to improve food security in remote Aboriginal and Torres Strait Islander communities^(23)^. The broader project and this framing study was co-designed by Aboriginal and Torres Strait Islander and non-Indigenous staff and nutrition, food security and policy academics and implemented, under the guidance of local Community Advisory Groups, in remote communities in Cape York and Central Australia.

The frames used in this study were informed by an earlier study that used street intercept interviews in two Australian capital cities to examine public support for policies to improve food security in remote Aboriginal and Torres Strait Islander communities^(24)^. We analysed the interview data to identify participants’ beliefs, values and the words and phrases they commonly used. From this analysis, we identified three distinct frames, each reflecting a different value orientation that prior research suggests is likely to shape attitudes toward public policy in positive ways^(25)^. A communication professional helped design the corresponding messages, which were intentionally brief and used similar language; they differed only in the key value being tested. Four messages were developed: a control message that was factual and had no values-based framing (Frame 1), a message focused on human rights (Frame 2), one appealing to fairness for all (Frame 3) and one emphasising that Aboriginal and Torres Strait Islander communities supported the solution (Frame 4). These messages were pilot tested and adjusted to ensure clarity and comprehension. The message statements are included in Table 1.

**Table 1.** Message frames used as stimulus material in survey

Frame	Message statement
Frame 1: Control	Right now, some Australians can’t afford to buy enough healthy food. This is a particular problem for Aboriginal and Torres Strait Islander people in remote communities. One reason for this is that people need to spend a lot of their income on electricity. Subsidising electricity in remote communities means people will have more income to put towards cost of living, including healthy food.
Frame 2: Human rights	As Australians, we believe that everyone should have basic human rights; however right now, some Australians can’t afford to buy enough healthy food. This is a particular problem for Aboriginal and Torres Strait Islander people in remote communities. One reason for this is people need to spend a lot of their income on electricity. Subsidising electricity in remote communities will help achieve this basic human right by allowing more income to be put towards cost of living, including healthy food.
Frame 3: Fairness	As Australians, we believe in fairness for all; however right now, some Australians can’t afford to buy enough healthy food. This is a particular problem for Aboriginal and Torres Strait Islander people in remote communities. One reason for this is that people need to spend a lot of their income on electricity. Subsidising electricity for remote communities is the fair thing to do as it will help level the playing field between cities and remote communities so they will have more income to put towards cost of living, including healthy food.
Frame 4: Community-identified solution	As Australians, we believe that everyone should have control over their future; however, right now some Australians can’t afford to buy enough healthy food. This is a particular problem for Aboriginal and Torres Strait Islander people in remote communities. One reason for this is that people need to spend a lot of their income on electricity. Leaders in remote communities have identified that subsidising electricity in their communities is a key solution as it will allow more income to be put towards cost of living, including healthy food.

In line with the study objective and previous research findings,^(25)^ we hypothesised that:

- Participants who support progressive parties (Greens or Labor) would be more likely to positively respond to the message promoting the policy option as community-identified (Frame 4).
- Participants who support progressive parties (Greens or Labor) would be more likely to positively respond to human rights framing (Frame 2).
- Participants who support conservative parties (Liberal, National or One Nation) would be more likely to positively respond to fairness framing (Frame 3).

### Sampling and data collection

Participants were recruited through an online panel from market research company Qualtrics to ensure a representative sample matching the demographics of the Australian population (in August 2023). The four messages, which included three messages with framing and one control (Table 1), were randomly assigned to participants, with even distribution of key characteristics including gender, age, education status and geographical area (urban *v*. regional/rural).

Political party preference was not adjusted for in the recruitment stage; however, the distribution in our sample was comparable to that reported in the Australian Electoral Study, which reflects the population preferences of Australian adults who voted in the 2022 federal election (Liberal National Coalition: 35·7 %, Labor: 32·6 %, Greens: 12·3 %, One Nation: 4·9 %)^(26)^. Therefore, the unweighted sample data were used in the following analyses.

### Variables of interest

The survey and variables used in this study are detailed in Supplementary file 1. The outcome variables were measured in two ways – ‘agreement with’ and ‘feeling towards’ an electricity subsidy – as differential intentions have been associated with each measure^(27)^. These variables were subdivided as measures of agreement.

Sociodemographic factors included gender, age, highest education level attained and geographical area (urban or regional/rural, as determined by the online panel company using participant postcodes). Additional sample characteristics included political party preference (Liberal/National/One Nation, Labor, Greens and Other [respondent supported independent candidates, was unsure or had no party preference]), and frequency of following the news (Table 2).

**Table 2.** Summary characteristics of nationally representative population sample

Characteristic	*N*	%
**Sex**		
Female	819	52·7
Male	726	46·7
Non-binary/third gender	6	0·04
Prefer not to say	4	0·03
**Age**		
18–24 years old	189	12·2
25–40 years old	466	30·0
41–64 years old	585	37·6
65 and over	315	20·3
**Education**		
Some secondary school or less	186	12·0
Finished secondary school	478	30·7
Diploma	347	22·3
University undergraduate	370	23·8
University postgraduate	174	11·2
**Area**		
Urban	985	63·3
Regional	567	36·5
Not sure	*3 missing observations*	*0·2*
**Political party affiliation**		
Labor	542	34·9
Liberal/National/One Nation	500	32·2
Greens	177	11·4
Undecided/Other*	336	21·6

* Participants who voted for independent candidates or stated they were unsure, undecided or had no political preferences.

### Data inclusion criteria

For survey data to be included, the respondent was required to:

- be an adult and Australian resident
- have consented to participate
- have spent more than 2·3 min (138 s) completing the online survey. This cut-off was recommended by the online panel company with an additional 10 % added.

### Data analysis

Logistic regression models were used to explore the associations between the outcome variables relating to support for an electricity subsidy and the message frames, as well as the sample characteristics. The adjusted OR (aOR; 95 % CI) of support for each outcome is presented. In the original models, Frames 2, 3 and 4 were compared to the control (baseline) frame. After observing the results, we also ran analyses which compared each frame to the frame with least agreement.

For the agreement question (‘How much do you agree with subsidising electricity in remote Aboriginal and Torres Strait Islander communities?’), the outcome used was strongly agree or agree (agreement) *v*. neither agree nor disagree, disagree or strongly disagree (no agreement). For the feeling towards the subsidy for electricity question (‘How do you feel about a subsidy for electricity in remote Aboriginal and Torres Strait Islander communities?’), the outcome was categorised as very positive or positive (positive feelings) *v*. neutral, negative or very negative (no positive feeling).

Interaction term analysis was conducted to explore whether the characteristics of age, gender, political party preference, education status or geographic area influenced responses to framed messages. To aid with interpretation, the distribution of respondents who were strongly or somewhat in support of the electricity subsidy (based on the agreement and positive feeling measures) was stratified by message framing blocks, and the significant interactions were highlighted.

## Results

The study participants were 1555 nationally representative adults across Australia (see Table 2). There were similar numbers of females and males. Two-thirds (67·6 %) were aged between 25 and 64, and 12 % were 18–24 years old. Thirty-five percent of respondents were university graduates. About one-fifth (21·6 %) said they supported independent political candidates, were unsure or had no party preference.

### Personal characteristics and levels of support for an electricity subsidy

Support for an electricity subsidy was higher among younger respondents (18–24 years), frequent news viewers and those with university qualifications. Respondents with a postgraduate degree showed higher odds of agreement (Table 3). Support also varied by political party preference, with high levels of agreement among Greens (75·1 %) and Labor party supporters (69·9 %), and lower levels from conservative (58·8 %) and ‘other’ (57·7 %) voters. A similar trend was observed for positive feelings towards the subsidy.

**Table 3.** Distribution of respondents in support of the electricity subsidy (*n* (%)) and the adjusted OR for each message framing block and demographic Table 3 long description.

	Agreement with subsidy				Positive feeling towards subsidy				Sub-group totals	
	*n*	%	aOR	95 % CI	*n*	%	aOR	95 % CI	*n*	%
Block	(*p* = 0·08)^†^				(*p* = 0·07)^‡^					
Frame 1: Control	255	64·7	baseline		198	50·3	baseline		394	25·3
Frame 2: Human rights	271	68·8	1·23	0·90, 1·67	216	54·8	1·23	0·92, 1·64	394	25·3
Frame 3: Fairness	228	60·2	0·82	0·61, 1·11	173	45·6	0·85	0·64, 1·14	379	24·4
Frame 4: Community	246	63·4	0·98	0·73, 1·33	208	53·6	1·16	0·87, 1·55	388	25·0
**Participant characteristics** ***Age groups***										
18–24 years	136	72·0	baseline		90	47·6	baseline		189	12·2
25–40 years	314	67·4	0·65*	**0·43, 0·96**	245	52·6	1·06	0·74, 1·52	466	30·0
41–64 years	359	61·4	0·54**	**0·36, 0·78**	303	51·8	1·14	0·80, 1·62	585	37·6
65 and over	191	60·6	0·55**	**0·35, 0·84**	157	49·8	1·13	0·75, 1·68	315	20·3
***Gender***										
Female	519	63·4	baseline		409	49·9	baseline		819	52·7
Male	473	65·2	1·00	0·80, 1·25	382	52·6	1·08	0·88, 1·33	726	46·7
Non-binary/other gender	4	66·7	1·28	0·24, 9·59	3	50·0	1·05	0·18, 6·26	6	0·04
Prefer not to say	4	100	NA		1	25·0	0·38	0·18, 3·33	4	0·03
***Education***										
Some secondary school or less	107	57·5	baseline		74	39·8	baseline		186	12·0
Finished secondary	288	60·3	0·92	0·64, 1·31	233	48·7	1·36	0·95, 1·94	478	30·7
Diploma	233	67·1	1·25	0·85, 1·83	169	48·7	1·27	0·88, 1·85	347	22·3
University undergraduate	236	63·8	1·00	0·68, 1·46	203	54·9	1·63*	**1·12, 2·39**	370	23·8
University postgraduate	136	78·2	1·93**	**1·19, 3·17**	116	66·7	2·49***	**1·59, 3·92**	174	11·2
***Area***										
Urban area	662	67·2	baseline		515	52·3	baseline		985	63·3
Regional or rural area	338	59·6	0·81	0·65, 1·01	280	49·4	1·00	0·80, 1·24	567	36·5
Not sure			NA						*3 missing obs*	
***Political party preference***										
Labor	379	69·9	baseline		305	56·3	baseline		542	34·9
Liberal/National/One Nation	294	58·8	0·60***	**0·46, 0·79**	228	45·6	0·61***	**0·48, 0·79**	500	32·2
Greens	133	75·1	1·17	0·79, 1·77	114	64·4	1·40	0·98, 2·03	177	11·4
Undecided/Other**^α^**	194	57·7	0·62**	**0·46, 0·84**	148	44·0	0·67**	**0·51, 0·89**	336	21·6
***Frequency of watching news***										
Hardly at all	62	48·1	baseline		46	35·7	baseline		129	8·3
Only now and then	174	59·0	1·50	0·97, 2·32	133	45·1	1·28	0·83, 2·00	295	19·0
Some of the time	452	68·0	2·30***	**1·54, 3·46**	351	52·8	1·78**	**1·19, 2·69**	665	42·8
Most of the time	312	67·0	2·21***	**1·44, 3·40**	265	56·9	2·00**	**1·31, 3·09**	466	30·0

* *p* < 0·05, **p < 0·01, ***p < 0·001; the control message frame was used as the baseline to which all the experimental frames (2–4) were compared to, while the baselines for all other measures that was used in logistic regression analyses are listed in the respective demographic. The *n* (%) values represent the somewhat and strong levels of support in each question for each subcategory.

^§^We did not adjust for variable in the multiple logistic regression model.

α The category ‘Undecided’/‘Other’ includes participants who voted for independent candidates or stated they were unsure, undecided or had no political preferences.

† A non-significant difference (*p* = 0·08) was found by the likelihood ratio test when the logistic regression model for agreement with all variables included was compared with a logistic regression model for agreement with all exposures minus the message frames variable.

‡ A non-significant difference (*p* = 0·07) was found by the likelihood ratio test when the logistic regression model for agreement with all variables included was compared with a logistic regression model for agreement with all exposures minus the message frames variable.

### Impact of message framing and support for the subsidy for electricity

The impact of message frames on participants’ level of support for the electricity subsidy was assessed against two different measures – agreement with the subsidy and positive feelings towards it (Table 3). Logistic regression analyses showed no statistically significant differences when comparing the three message frames to the control. Overall, participants who received Frame 3 (fairness) showed the lowest levels of agreement with or positive feelings toward the subsidy. Conversely, those who received Frame 2 (human rights) had the highest (68·8 % *v*. 64·7 % in the control and 54·8 % *v*. 50·3 % in the control, respectively).

### Comparing message frames

Using Frame 3 (fairness) as the baseline category, we found that participants who received Frame 2 (human rights) had 46 % greater odds of agreement with and 44 % greater odds of positive feelings towards the subsidy (Supplementary file 2). The community support framing (Frame 4) was also associated with 38 % higher odds of support for the subsidy compared to the fairness frame (Frame 2). These differences were statistically significant.

### Interaction between exposure variables and outcome variables

#### Political preference

We hypothesised that progressive political party supporters would be more likely to positively respond to the community-identified solutions frame (Frame 4) and the human rights frame (Frame 2). While both these frames increased the odds of agreement with the electricity subsidy compared to the control (by 23 % for Frame 4 and 31 % for Frame 2), the interactions were not statistically significant. We also hypothesised that conservative party supporters were more likely to respond to the fairness frame (Frame 3). However, no significant interaction was observed (Table 4).

**Table 4.** Distribution of respondents in support of the electricity subsidy (*n* (%)) stratified by message framing blocks and moderation analysis interaction results

	Agreement with subsidy								Positive feeling towards subsidy							
Participant characteristics	Frame 1: Control		Frame 2: Human rights		Frame 3: Fairness		Frame 4: Community		Frame 1: Control		Frame 2: Human rights		Frame 3: Fairness		Frame 4: Community	
**Political party**	ns	ns														
Labor	101	69·2	94	74·6	90	63·4	94	73·4	84	57·5	78	61·9	70	49·3	73	57·0
Liberal/National/One Nation	74	57·8	78	61·9	64	57·1	78	58·2	53	41·4	61	48·4	46	41·1	68	50·7
Greens	35	81·4	39	81·2	**23**	59·0*	36	76·6	28	65·1	34	70·8	19	48·7	33	70·2
Undecided/Other^†^	45	58·4	60	63·8	51	59·3	38	48·1	33	42·9	43	45·7	38	44·2	34	43·0
**Geographical area**	ns	ns														
Urban	158	64·0	**195**	73·6*	155	64·6	154	66·1	125	50·6	151	57·0	111	46·3	128	54·9
Regional or rural	97	66·0	76	58·9	**73**	53·3*	92	59·7	73	49·7	65	50·4	62	45·3	80	51·9
**Age**	ns	ns														
18–24 years	24	63·2	**46**	88·5**	37	68·5	29	64·4	18	47·4	25	48·1	24	44·4	23	51·1
25–40 years	90	69·2	81	68·6	73	64·0	70	67·3	71	54·6	70	59·3	49	43·0	55	52·9
41–64 years	90	59·6	96	62·7	80	60·6	93	62·4	74	49·0	84	54·9	66	50·0	79	53·0
65 and over	51	68·0	48	67·6	**38**	48·1*	54	60·0	35	46·7	37	52·1	34	43·0	51	56·7
**Gender**	ns	ns														
Female	136	64·2	140	69·3	117	56·8	126	63·3	104	49·1	109	54·0	90	43·7	106	53·3
Male	118	65·2	129	67·9	108	64·3	118	63·1	93	51·4	106	55·8	82	48·8	101	54·0
**Education**	ns	ns														
Some secondary	29	59·2	27	62·8	25	52·1	26	56·5	17	34·7	20	46·5	15	31·3	22	47·8
Finished secondary	57	55·3	79	68·1	71	55·0	81	62·3	43	41·7	**64**	55·2*	63	48·8	63	48·5
Diploma	70	72·2	66	70·2	52	62·7	45	61·6	45	46·4	51	54·3	36	43·4	37	50·7
University undergraduate	60	61·2	70	70·0	46	61·3	60	61·9	54	55·1	56	56·0	36	48·0	57	58·8
University postgraduate	39	83·0	29	70·7	34	77·3	34	81·0	39	83·0	**25**	61·0*	**23**	52·3**	29	69·0

Significant (*p* < 0·05) and non-significant differences found by the likelihood ratio rest to compare original and interaction-term models.

* *p* < 0·05, significant interaction was present in the subcategory.

**p < 0·01, significant interaction was present in the subcategory.

*Note*: the ‘not sure’ category for geographical area was not presented in this table as there were no meaningful findings due to the small sample size.

† The category ‘Other’ includes participants who voted for independent candidates or stated they were unsure, undecided or had no political preferences.

Although we did not find a significant interaction between political preference and message frame, there was a notable exception. Greens supporters who received Frame 3 (fairness frame) were less likely to agree with the subsidy than those in the control group (Table 4, 59·0 % v 81·4 %). In addition, the lowest level of agreement was observed among participants in the other/undecided political group who received the community-identified solutions frame (Frame 4, 48·1 %).

#### Geographical area

Overall, we found no strong evidence that geographic location moderated the effect of the message frames, although some differences were observed. Participants living in urban areas who received Frame 2 (human rights frame) had 57 % higher odds (*p* < 0·05) of agreeing with the subsidy. Regional or rural participants who received Frame 3 (fairness frame) had 41 % lower odds of agreeing with the subsidy compared to the control frame.

#### Age

While the overall likelihood ratio tests between frame and age were not statistically significant, notable differences emerged in specific categories. Younger respondents (18–24 years) who received the human rights frame (Frame 2) were significantly more likely to agree with the electricity subsidy compared to the control frame (88·5 % v 63·2 %). In contrast, participants aged 65 and over who received the fairness message frame (Frame 3) had 56 % lower odds of agreeing with the subsidy (Table 4).

#### Education

Among participants who finished secondary school, Frame 2 (human rights) increased the odds of positive feelings by 72 % compared with the control. In contrast, respondents with postgraduate degrees who received Frame 2 (human rights) and Frame 3 (fairness) were significantly less likely to report positive feelings towards the subsidy, by 68 % and 78 %, respectively (Table 4).

#### Gender

Overall gender did not significantly influence support for the electricity subsidy across message frames. However, for females, Frame 3 (fairness) decreased the odds of agreement with and positive feelings towards the electricity subsidy (Table 4).

## Discussion

This experimental framing study was designed to explore whether message framing influences individuals’ support for policy actions to improve food security in remote Aboriginal and Torres Strait Islander communities. We also examined whether political affiliation and sociodemographic factors affected support for a specific policy, an electricity subsidy, when message frames were introduced, and the impacts of different frames.

### Effects of message framing

In examining the effects of message frames on support for the electricity subsidy, no clear differences were found in terms of agreement with or positive feelings towards the subsidy between the experimental frames and the control frame. However, when considering specific frames, the group receiving the fairness frame (Frame 3) demonstrated the lowest level of support, while the human rights frame (Frame 2) generated the highest level, although the difference was marginal.

Interestingly, when the fairness frame (Frame 3) was used as the baseline in place of the control frame, we observed some statistically significant outcomes. Participants who received the human rights frame (Frame 2) showed higher agreement with and positive feelings towards an electricity subsidy than those who received the fairness frame. Similarly, participants who received the community message frame (Frame 4) showed greater positive feelings towards the subsidy than recipients of the fairness frame. This suggests that messages rooted in human rights or community-led values may resonate more deeply with individuals than an appeal to fairness, especially in the context of this policy solution.

The limited impact of the message frames in our study may have been moderated by participants’ pre-existing policy preferences^(28)^. Previous studies have found that personal characteristics and political ideology of respondents may be better predictors of support for policies than message frames^(28,29)^. Consistent with these findings, respondents who supported the Greens, had postgraduate degrees or watched the news more frequently showed higher levels of agreement with the electricity subsidy regardless of the frame they received. Individuals with these characteristics typically support public health policies^(30)^, and their responses may reflect a predisposition toward policies like an electricity subsidy rather than responsiveness to framing.

### Interactions of message frames, political preferences and sociodemographic factors

We expected that framing messages to emphasise community-identified solutions to food insecurity would increase support for the proposed policy. Although some interactions between message frames and political preferences were observed, overall support for the electricity subsidy did not vary notably by political preference or sociodemographic factors. This may reflect the low salience of remote food security for many participants, resulting in a weak connection to their political or social identities, and consequently limited engagement and shallow processing of the message^(27)^.

Specifically, there were similar levels of support for the subsidy among progressive party supporters who received the community, human rights and control message frames. Contrary to our hypotheses, the community and human rights frames did not significantly bolster support for the subsidy among progressive voters. This may be due to a ceiling effect, where the majority of participants already agree with a proposed policy, leaving little room for further influence^(31)^. This was particularly evident among Greens voters who generally supported the electricity subsidy more than participants from other political parties, when measured *without* message frames. The lack of significant variation in support across frames suggests that framing may not significantly change outcomes when the majority of participants are already in favour of the policy.

One of the most intriguing findings of this study was the negative impact of the ‘fairness’ frame. For example, Greens supporters who received the fairness frame were significantly *less* likely to agree with the electricity subsidy compared to those who received the control frame. This result is particularly surprising, given that Greens voters typically support policies that emphasise fairness and equity^(32)^. Additionally, the fairness frame was associated with reduced support among regional participants, women and older respondents, suggesting that framing effects may vary across different demographic groups. Further, and contrary to our hypothesis that conservative voters who received the fairness frame would be more likely to support the subsidy, it had no effect compared to the control frame. These findings are notable because fairness is often cited as a universal value recommended for promotion in policy advocacy communications^(25)^. In contrast, our findings suggest that the fairness frame did not have broad appeal in messaging about an electricity subsidy as a policy solution to improve food security. It may be that fairness does not carry the same appeal in this context, potentially due to its overuse in political discourse^(33)^ or a perception that the subsidy does not represent a ‘fair distribution of resources’^(34)^. More research is necessary to test the validity of the assumption that the fairness frame has universal appeal in policy debates.

It is also important to note that while agreement with and positive feelings toward the electricity subsidy generally followed the same trend, there were some notable exceptions. For instance, younger participants (18–24 years) who received the human rights frame (Frame 2) reported higher agreement with the subsidy but did not show a corresponding increase in positive feelings. This discrepancy suggests that individuals may be more likely to express agreement with a policy than to feel positively about it, particularly when the framing emphasises moral or rights-based arguments. This distinction between agreement and emotional response could be further explored in future studies, especially given the observed difference in younger participants’ responses to the human rights frame.

Another finding with implications for advocates was the lower level of support for the subsidy among undecided and ‘other’ voters when the ‘community-identified solutions’ frame (Frame 4) was used. This suggests that, for some audiences, community-driven solutions may be perceived as divisive or exclusive. Previous research has shown that while some groups in society embrace community-led approaches, others – particularly those with limited trust in government action or weak connection to affected communities – may view such solutions with scepticism^(35,36)^. This may be especially relevant where voters do not feel connected to, or well-informed about, Aboriginal or Torres Strait Islander communities. Additionally, some voters may believe that an electricity subsidy should be available to all Australians, not just remote communities. This sentiment was also evident in the first qualitative phase of this research^(24)^. Given that undecided voters are an electorally pivotal cohort frequently targeted in campaigns, message strategies may need to be adapted for this group.

### Policy support and political party preference

We found a strong association between political party preference and support for the electricity subsidy regardless of message framing. Participants who were identified as conservative or undecided voters had the lowest levels of support for the subsidy in contrast to progressive party supporters. This mirrors other studies of public opinion towards a range of public health policies that target the broad population^(37)^. This finding may be because individuals’ political ideological tendencies influence their positions on policy issues and their voting behaviour^(38)^. As undecided voters are often a crucial cohort in election outcomes and hence the targets of election campaigns^(39)^, more research is needed to determine the most effective message frame to gain support from this cohort and put this issue on the policy agenda.

### Implications for advocacy and policy communication

Although this study focused on a specific policy response to food security in remote Aboriginal and Torres Strait Islander communities, the findings have broader relevance for nutrition advocacy and policy communication. In particular, they highlight the limited and context-dependent impact of values-based framing on public support for nutrition-related policies. Across nutrition policy domains, framing is often promoted as a key strategy for building public support^(2)^. However, our findings suggest that framing effects are modest and were outweighed by pre-existing political orientation, education and news engagement. This underscores the importance of advocates using audience segmentation and message testing, rather than reliance on universally recommended frames.

The weaker performance of fairness-based framing is particularly instructive for nutrition advocates, as fairness is commonly invoked in debates around food affordability, regulation and access to healthy diets^(24)^. These results suggest that such frames may not resonate uniformly and may, in some cases, reduce support among particular groups.

### Strengths and limitations

A key strength of this study is the large number of participants and the use of a demographically representative sample, enhancing the generalisability of the findings.

However, the following limitations should be acknowledged. First, the primary outcome was an immediate measure of attitudes towards the policy, which may not reflect long-term opinion change. Second, the study presented only supportive messages, whereas in real-world settings, individuals are often exposed to conflicting messages about policy issues. Future research should explore how message framing interacts with competing information. Third, a large number of statistical tests were conducted, raising the possibility of false positive results. While this was partly mitigated by predefining hypotheses, it is important to note that two of those were based on limited prior evidence.

## Conclusion

This experimental study found that message framing had limited impact on support for an electricity subsidy as a policy solution to help improve food security in remote Aboriginal and Torres Strait Islander communities. Although the human rights frame elicited the highest support and the fairness frame the lowest, this effect was generally limited and often moderated by participants’ pre-existing preferences, political affiliations and sociodemographic factors. The findings point to the need for more research on targeted framing strategies, particularly for undecided or swing voters, who may be more responsive to framing than those with entrenched views. Further research is needed to explore why certain frames, like the fairness frame, did not yield the expected effects, particularly among groups such as Greens voters and older participants. Additionally, investigating the nuances between agreement and emotional responses to policies could offer a deeper understanding of how different groups process policy messages.

## Supporting information

10.1017/S1368980026102845.sm001 Katta et al. supplementary material Katta et al. supplementary material

## Supplementary material

For supplementary material accompanying this paper visit https://doi.org/10.1017/S1368980026102845

## Table 3. Long description

The table presents data on the support for an electricity subsidy among various demographic groups and message framing blocks. It includes columns for agreement with the subsidy, positive feelings towards the subsidy, and sub-group totals. The table is divided into several sections: Block, Participant characteristics (Age groups, Gender, Education, Area, Political party preference, Frequency of watching news), and Sub-group totals. Each section contains rows with specific categories and their corresponding values. The table provides the number of respondents (n), percentage (%), adjusted odds ratio (aOR), and 95% confidence interval (CI) for each category. Notable trends include higher support among younger respondents, frequent news viewers, and those with university qualifications. Support also varies by political party preference, with higher levels among Greens and Labor party supporters, and lower levels from conservative and other voters.

Navigate back to Table 3..
