# Supplementary material for: Advocacy to improve food security in remote Aboriginal and Torres Strait islander communities in Australia: measuring the impact of message frames
Source: Public Health Nutr. 2026 Jun 3;29(1):e127. doi: 10.1017/S1368980026102845 (PMC13434583; doi:10.1017/S1368980026102845)
Supplement: Supplementary file 1 [file S1368980026102845sup001.docx]

**Supplemental file 1: Description of survey and response questions**

| **Concept** | **Survey question prompt** | **Response options** | **Use in logistic regression and overall analysis** |
| --- | --- | --- | --- |
| Age | Are you 18 years or over? | Yes | Screening for survey participation eligibility; included |
|  |  | No | Excluded |
| Citizenship | Are you an Australian resident? | Yes | Screening for survey participation eligibility; included |
|  |  | No | Excluded |
| Consent |  | Yes, I agree to participate in this study. | Screening for survey participation eligibility; included |
|  |  | No, I do not agree to participate in this study. | Excluded |
| Agreement with subsidy | How much do you agree with subsidising electricity in remote Aboriginal and Torres Strait Islander communities? | Strongly agree,  Somewhat agree | Agreement |
|  |  | Neither agree nor disagree,  Somewhat disagree,  Strongly disagree | No agreement |
| Feelings towards subsidy | How do you feel about a subsidy for electricity in remote Aboriginal and Torres Strait Islander communities? | Very positive,  Positive | Positive feelings |
|  |  | Neutral,  Negative,  Very negative | Neutral or negative feelings |
| Gender | What is your gender? | Male,  Female,  Non-binary/ third gender,  Prefer not to say |  |
| Age | Age range: | 18 – 24 years,  25 – 40 years,  41 – 64 years,  65 and over |  |
| Postcode | Please write your postcode in the text box. | Free-text response |  |
| Education | What's the highest level of education you have completed? | Some secondary school or less,  Finished secondary school, Diploma,  University undergraduate degree,  University postgraduate degree |  |
| Political party | Which political party would you usually vote for? | Not sure,  Liberal Party,  Labor Party,  National Party,  Greens Party,  One Nation Party, Independent,  Other (please state) |  |
| Frequency of thinking about food security for Australians | In the past 12 months, how often have you thought about how food affects the health of Australians? | 2-3 times per week,  Once a week,  Once a month,  Once every 6-12 months, Never |  |
| Frequency of following news | Some people follow what's going on in government and the news most of the time. Others aren't that interested. Would you say you follow what's going on in government and the news: | Hardly at all,  Only now and then,  Some of the time,  Most of the time |  |
| Regionality | Metro  Regional |  |  |
| Message Frames | Frame 1: FL_80  Frame 2: FL_81  Frame 3: FL_82  Frame 4: FL_83 |  |  |

**Supplemental file: Comparing the message frames with Frame 3 as baseline**

| **Demographics** | **Q17 Agreement with subsidy** | | **Q19 Positive feeling towards subsidy** | |
| --- | --- | --- | --- | --- |
|  | **n (%)** | **OR (95% CI)** | **n (%)** | **OR (95% CI)** |
| Frame 1 (control frame) | 255 (64.7) | 1.21 (0.91, 1.63) | 198 (50.3) | 1.20 (0.91, 1.60) |
| Frame 2 (human rights) | 271 (68.8) | **1.46 (1.09, 1.96)*** | 216 (54.8) | **1.44 (1.09, 1.92)*** |
| Frame 3 (fairness) | 228 (60.2) | baseline | 173 (45.6) | baseline |
| Frame 4 (community-identified solution) | 246 (63.4) | 1.15 (0.86, 1.54) | 208 (53.6) | **1.38 (1.04, 1.83)*** |
